# pH-driven conformational switch between non-canonical DNA structures in a C-rich domain of *EGFR* promoter

**DOI:** 10.1038/s41598-018-37968-8

**Published:** 2019-02-04

**Authors:** Camilla Cristofari, Riccardo Rigo, Maria Laura Greco, Michele Ghezzo, Claudia Sissi

**Affiliations:** Department of Pharmaceutical and Pharmacological Science, Padova, 35131 Italy

## Abstract

EGFR is an oncogene that encodes for a trans-membrane tyrosine kinase receptor. Its mis-regulation is associated to several human cancers that, consistently, can be treated by selective tyrosine kinase inhibitors. The proximal promoter of EGFR contains a G-rich domain located at 272 bases upstream the transcription start site. We previously proved it folds into two main interchanging G-quadruplex structures, one of parallel and one of hybrid topology. Here we present the first evidences supporting the ability of the complementary C-rich strand (EGFR-272_C) to assume an intramolecular i-Motif (iM) structure that, according to the experimental conditions (pH, presence of co-solvent and salts), can coexist with a different arrangement we referred to as a hairpin. The herein identified iM efficiently competes with the canonical pairing of the two complementary strands, indicating it as a potential novel target for anticancer therapies. A preliminary screening for potential binders identified some phenanthroline derivatives as able to target EGFR-272_C at multiple binding sites when it is folded into an iM.

## Introduction

Epidermal growth factor receptor (EGFR) is a 170 kDa transmembrane oncoprotein formed by an intracellular catalytic tyrosine kinase domain, a single hydrophobic transmembrane helix and an extracellular N-terminal domain. The latter represents the recognition element for at least seven different endogenous ligands (i.e. EGF, TGF-α and HB-EGF) that induce EGFR dimerization and auto-phosphorylation. This EGFR activation switches on multiple pathways promoting cell proliferation, survival, adhesion and differentiation^1–4^. Consequently, EGFR overexpression and/or mutations are frequently associated to the pathogenesis and progression of several human cancers, such as non-small cell lung, breast or colon cancer^5^. The currently available therapeutics to counteract the upregulation of EGFR are based on the use of tyrosine kinases inhibitor (i.e. Gefitinib, Erlotinib) and humanized monoclonal antibodies (i.e. Cetuximab, Panitumumab and Necitunumab)^6–8^. Although these drugs are extremely efficient, they induce fast and frequent selection of resistant cells thus making the therapy ineffective.

A strategy to overcome these drawbacks lies in impairing the production of the protein. Different approaches have been exploited to achieve this goal. One refers to the use of short synthetic oligonucleotides properly designed to target, preferentially, the mRNA (i. e. antisense, siRNA, miRNA)^9,10^. Alternatively, a fine tuning of protein expression can be obtained by controlled modifications of the conformational features of gene promoters^11,12^. Indeed, although the B-form is the predominant DNA secondary structure within the cell, several studies support that biological processes as transcription and translation can be regulated also by other non-canonical DNA conformations (i. e. Z-DNA, A-DNA, hairpin, triple- or tetra-helices) which occur under unique environmental conditions and/or at specific nucleic acid sequences^13,14^. Thus, they might represent novel targets for the treatment of diseases associated to the aberrant expression of selected proteins.

Among the various non-canonical structures adopted by nucleic acids, large attention has been given to tetra-helices such as G-quadruplex (G4) and i-Motif (iM), formed by guanine-rich and cytosine-rich sequences, respectively. As far it concerns G4, four strands of variable polarity are held together by co-planar pairing of four guanines to generate G-tetrads that stack one over the other^15^. Conversely, iM is formed by two parallel duplexes arranged according to an antiparallel orientation one to each other^16–18^. The building block of this structure is a C-C^+^ base pair supported by Hoogsteen hydrogen bonds in which one cytosine must be protonated at N3. It derives that in general iM formation is favoured at pH close to the cytosine pKa (pKa 4.6). However, several contributions (i.e. the number of involved cytosines, the type of bases adjacent to the C-rich portion, organization of loops, the presence of proteins or ligands) can significantly increase the stability of these structures up to physiologically relevant pH^19–23^. Nowadays, the prediction of possible implications of iM in biological processes, has been finally supported by in-cell NMR spectroscopy and imaging with the use of a selective antibody^24,25^.

Recently we showed that *EGFR* promoter contains a G-rich sequence located 272 bases upstream the transcription start site (EGFR-272, 56.6% of guanines) that is able to fold into G4^26^. Clearly, the presence of such a G-rich portion implies the presence of a complementary C-rich strand where iM formation can be envisaged^27–29^. Here we present the first evidences supporting the ability of this C-rich sequence (EGFR-272_C) to assume an iM structure. We showed that this tetra-helical conformation is significantly stable but, according to the experimental conditions (pH, presence of cosolvent or salts and small molecules) it can be converted into an intramolecular arrangement likely referring to a hairpin. Interestingly, the iM efficiently competes with the canonical Watson and Crick pairing of the two complementary strands of *EGFR* promoter thus pointing toward a potential role of this iM in the regulation of EGFR transcription. This prompted us to explore its targeting by small molecules. As potential binders we tested some phenanthroline (Phen) derivatives that preferentially generate mono- or bis-Phen Ni(II) complexes according to the substitution pattern of the aromatic core. The interest in these systems derived by the fact that each single species was previously confirmed to be endowed with peculiar selectivity profiles towards different DNA conformations^30–33^. Overall, we confirmed that a single Phen unit is sufficient to obtain derivatives able to remarkably increase the stability of the iM folding of EGFR-272_C.

## Results and Discussion

### EGFR-272_C folds into an i-Motif structure

**Figure 1:**
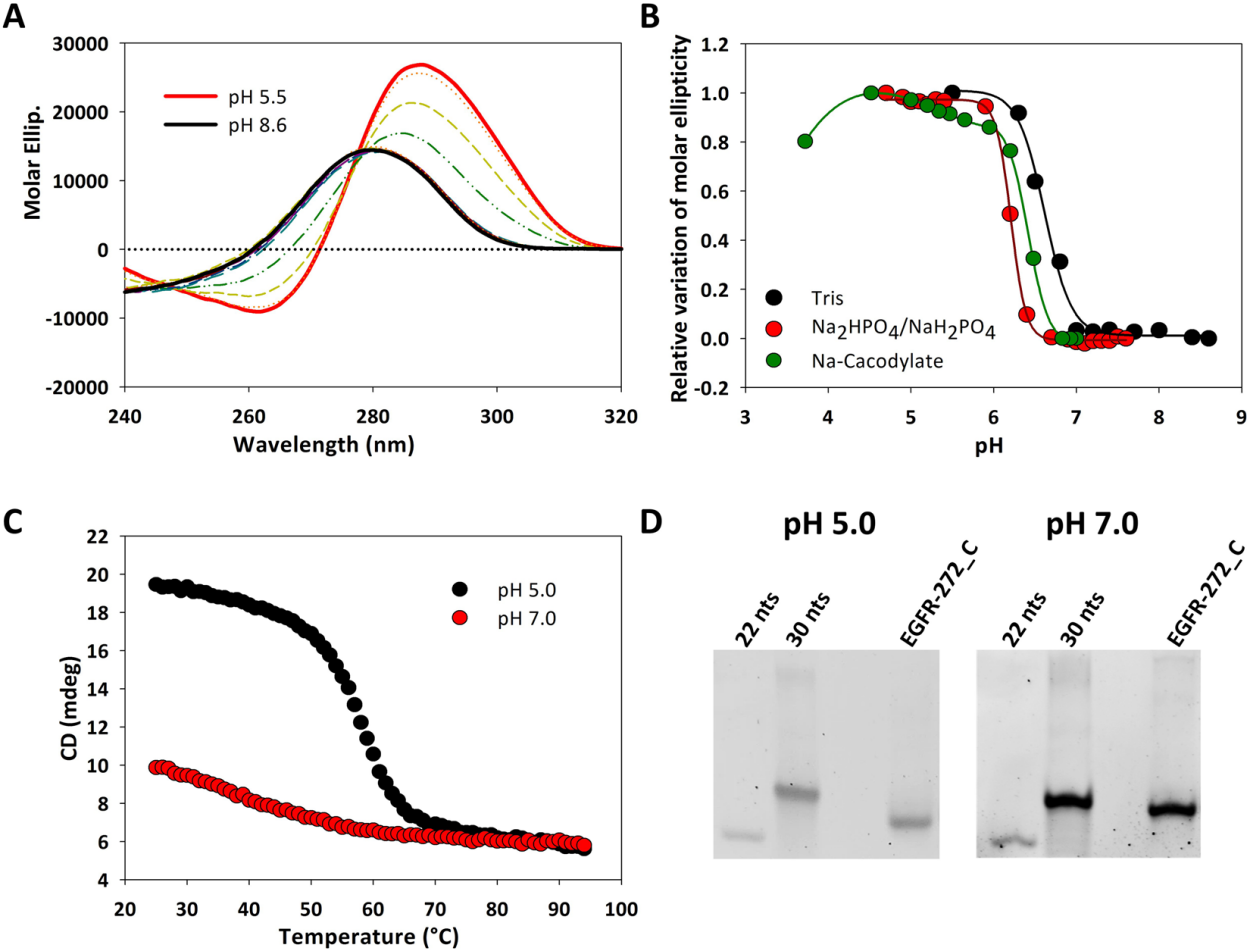
EGFR-272_C folds into a stable iM structure in acidic conditions. (**A**) CD spectra of 4 µM EGFR-272_C recorded at pH ranging from 5.5 to 8.6 in 10 mM Tris at 25 °C. (**B**) Relative variation of molar ellipticity at 288 nm as a function of pH recorded in different buffers. (**C**) Variation of the CD signal of 4 µM EGFR-272_C recorded at 288 nm in 10 mM Na-Cacodylate at pH 5.0 (black dots) and 7.0 (red dots) as a function of the temperature. (**D**) EGFR-272_C annealed at pH 5.0 and 7.0 and solved by PAGE in 1x TAE or 1x TBE, respectively. 22 nts and 30 nts refer to oligonucleotides unable to form intramolecular secondary structures under the tested conditions. Full-length gels are presented in Supplementary Fig. S1.

As previously anticipated, iM structures formation requires the hemi-protonation of cytosine residues. At the same time, buffer composition might influence nucleic acids folding. Thus, the folding behaviour of EGFR-272_C has been explored in a wide range of pH using three different buffers (10 mM Tris, Na-Cacodylate or Na_2_HPO_4_/NaH_2_PO_4_). At pH 4.5–5.5, a condition compatible with cytosine hemi-protonation, the dichroic profile of EGFR-272_C was characterized by a strong positive peak at 288 nm and a negative one centred at 240 nm that matches with the presence of an iM structure (Fig. 1A)^18,34,35^. As expected for this nucleic acid arrangement, at the lowest tested pH (pH = 3.5), where cytosines are fully protonated, the fraction of folded oligonucleotide dropped (Fig. 1B)^36^. Consistently, also the increment of pH caused a significant decrease of the CD signal at 288 nm as a result of cytosine deprotonation and, concurrently, iM unfolding. Nevertheless, the optical contribution of the iM was preserved up to pH 6.5. This folding profile appears to be shared in the different tested buffers. Noteworthy, 10 mM Tris keeps the iM arrangement at slightly higher pH values (Fig. 1B).

The thermal stability of EGFR-272_C at pH 5.0 and 7.0 was investigated by monitoring the CD signal variation at 288 nm as a function of the temperature (25–95 °C) (Fig. 1C). In acidic condition, the denaturation process showed a single perfectly reversible melting transition with T_m_ = 58.0 °C. The thermodynamic analysis of the denaturation curve provided an unfolding enthalpy ΔH = −76.93 kcal/mol. According to a previously reported model^37^, this value is consistent with the formation of about seven C-C^+^ pairs, as expected by the pairing of all the cytosines located in the four C-runs, or with a mixture of C-C^+^ and Watson-Crick base pairs.

Indeed, also at pH 7.0 a significant variation of the CD signal according to the temperature increment was recorded. Thus, we inferred that, as already reported for the complementary G-rich strand, EGFR-272_C might fold into an intramolecular hairpin-like arrangement in these conditions^22^. Native PAGE experiments supported this hypothesis (Fig. 1D). In fact, at pH 7.0, EGFR-272_C showed higher electrophoretic mobility than an oligonucleotide of the same number of residues but unable to assume any stable intramolecular secondary structure. At pH 5.0 the electrophoretic mobility of EGFR-272_C was further increased to a little extent. Interestingly, PAGE experiments did not highlight formation of any multimeric species. In agreement, CD spectrum of EGFR-272_C acquired at pH 5.0 was not affected by increasing oligonucleotide concentration (Supplementary Fig. S2). All these evidences point out that EGFR-272_C folds into an intramolecular iM in a slightly acidic environment.

### Watson-Crick base pairs within the loops favourably contribute to EGFR-272_C iM stability

According to the pH, EGFR-272_C can shift between a hairpin and an intramolecular iM. To better clarify the occurrence of these two folding states and to identify the paired residues that support these two arrangements, we performed S1 footprinting assay. S1 is a nuclease which selectively cleaves at unpaired nucleotide as found in single stranded DNA, loops and bulges. This peculiar activity makes it a suitable enzymatic probe for mapping loops in complex structures, as iM or hairpin^38^. To appreciate their localization in the different structures of EGFR-272_C, we performed the assay at pH 5.5 and pH 7.0.

**Figure 2:**
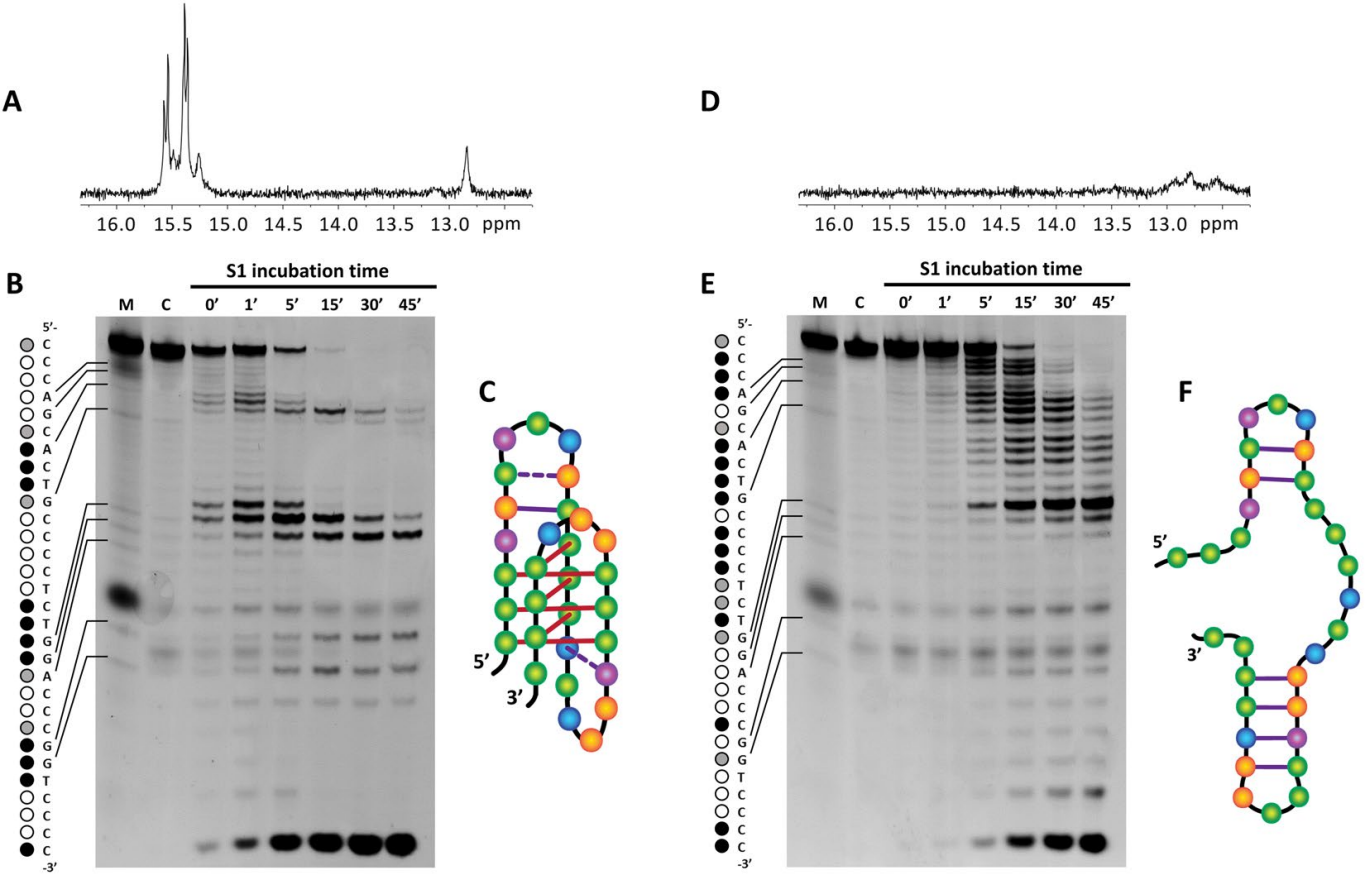
EGFR-272_C folds into different intramolecular structures according to the pH of the environment. (**A**–**D**) Imino region of ^1^H NMR spectrum of 0.15 mM EGFR-272_C acquired at 25 °C in 20 mM Na_2_HPO_4_/NaH_2_PO_4_ at pH 5.5 (**A**) and pH 7.0 (**D**) on a 600 MHz NMR spectrometer. (**B**–**E**) S1 footprinting of EGFR-272_C in 10 mM Na-Cacodylate at pH 5.5 (**B**) and 7.0 (**E**). On the left side, dots indicate the cleavage sites (black, grey and white dots refer to strong cleavage, moderate cleavage and not cleaved residues, respectively). M refers to Maxam and Gilbert purine marker (purine residues are indicated by arrows on the left side). (**C**–**F**) Proposed topology adopted by EGFR-272_C at pH 5.5 (**C**) and 7.0 (**F**).

Upon incubation of EGFR-272_C with S1 nuclease in acidic conditions, most cytosines were extensively protected from enzymatic digestion whereas three sets of cleavage sites were clearly mapped (Fig. 2B). They presented three or four strong cleavage bands each that must be associated to not paired residues. Consistently with their localization in EGFR-272_C, we identified them as the loops of the iM. In particular, looking at the residues more prone to be digested by the nuclease, we related A7-C8-T9 to the first loop, C16-T17-G18-G19 to the second and G24-G25-T26 to the third one. Noteworthy, the enzymatic processivity of S1 was significantly reduced at some specific sites which are not part of the iM core, as G5, C6, G10, C11, T15 and A20. To fold into an iM, the oligonucleotide requires to align its C-runs in antiparallel orientations and this makes possible to localize G5**·**C11, C6**·**G10, T15**·**A20 at positions suitable for Watson-Crick base pairing. Among them, the stronger protection was observed for G5**·**C11. A closer look at the C-runs revealed that four stretches of cytosines (C1-C3, C12-C14, C21-C23, C27-C29) were completely resistant to S1 cleavage, supporting they are involved in six C-C^+^ base pairs. Conversely, C30 was associated to a strong cleavage site: this suggests that it is not engaged in any base pairing but it freely protrudes into solution. In agreement with these findings, ^1^H NMR spectrum of EGFR-272_C recorded at pH 5.5 showed a cluster of six peaks between δ 15.20 and 15.60 ppm (Fig. 2A) which are consistent with cytosine imino protons characteristic of hemi-protonated C-C^+^ base pairs. Moreover, an imino peak at δ 12.83 ppm indicated the presence of a stable G**·**C base pair in Watson-Crick geometry. This signal might be associated to G5·C11 base pair which is expected to be the most stable Watson-Crick pairing in the structure. The exposure and flexibility of external C6**·**G10 and T15·A20 base pairs make their detection difficult. From these data we derived the model of EGFR-272_C iM reported in Fig. 2C.

At neutral pH, S1 caused an extensive cleavage of the oligonucleotide as expected for a predominantly single-stranded sequence. Nevertheless, few residues appeared more protected and other as preferential cleavage sites. This pattern cannot be explained considering a single well-defined hairpin conformation, but rather a pool of fold-back arrangements may be envisaged. Based on the intensity of the electrophoretic bands, T17 and C30 were identified as the most significant cleavage sites: this indicates that they are not paired. Conversely, C11 and G5 seem to be the most protected residues within an almost completely cleaved stretch. EGFR-272_C sequence was then submitted to an *in silico* structural prediction tool (IDT-Oligoanalyzer) and the resulting outputs (4 hits) were screened to fit the S1 nuclease protection pattern. A single fold, reported in Fig. 2F, resulted compatible with the experimental evidences. In addition, NMR spectrum of EGFR-272_C acquired at pH 7.0 showed at least three peaks in the imino regions corresponding to canonical G-C base pairs (Fig. 2D). They were poorly resolved but this is in agreement with the low stability of the predicted hairpin in these experimental conditions. Thus, the reported model likely represents the most stable hairpin form assumed by EGFR-272_C in solution at pH ≥ 7.0.

### Solution composition affects the conformational equilibria of EGFR-272_C

Formation and stability of DNA secondary structures can be largely affected by ionic composition and crowding condition within cell. Thus, in order to mimic physiological environment, we explored the conformational equilibria of EGFR-272_C in the presence of K^+^ ions, which are the most abundant cationic species in the cell, and in the presence of polyethylene glycol (PEG), a widely used crowding agent.

**Figure 3:**
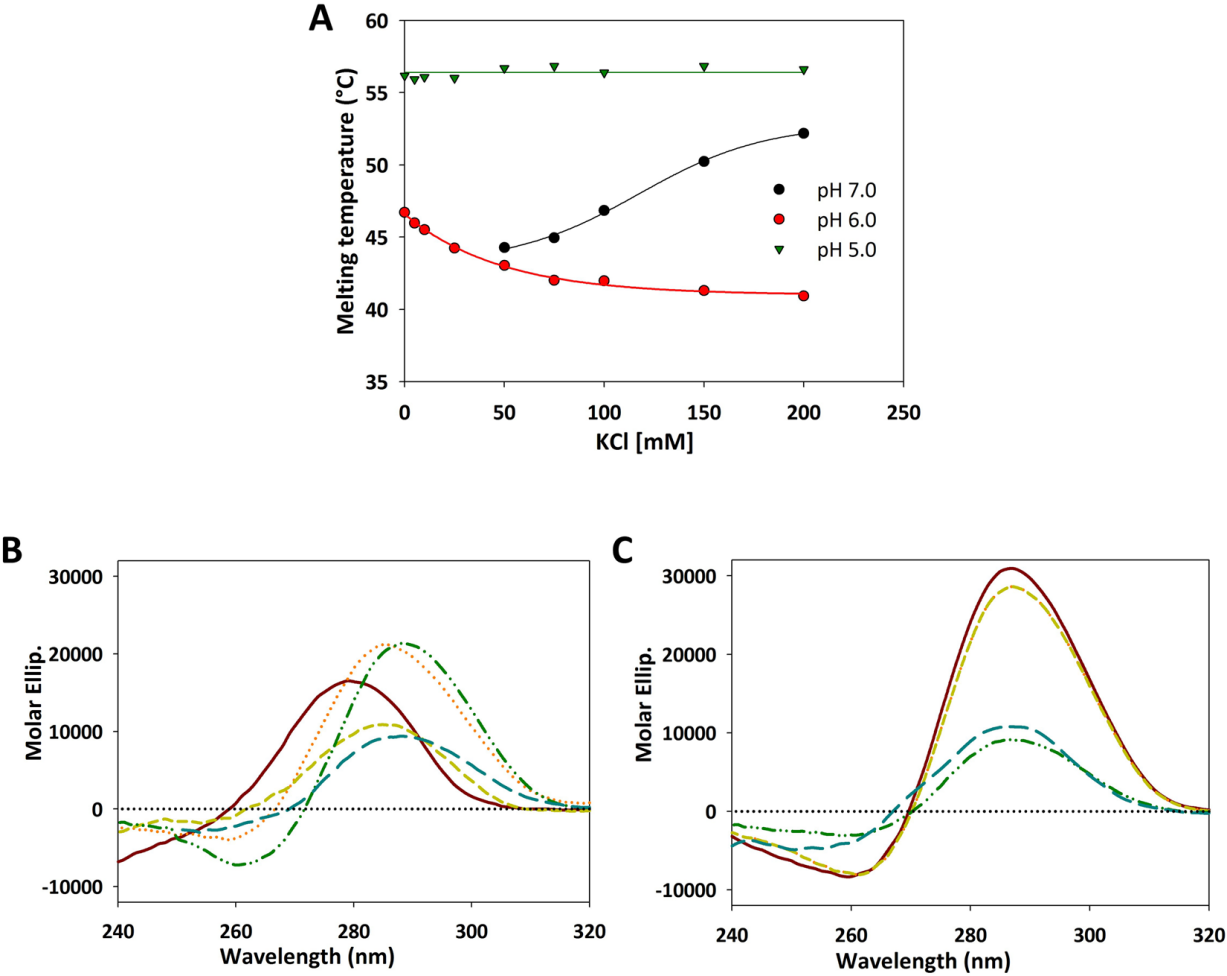
The interaction of KCl and PEG with EGFR-272_C is a function of pH. (**A**) Melting temperature of 0.25 μM *EGFR-272_C recorded in 10 mM Na_2_HPO_4_/NaH_2_PO_4_ (pH 6.0 and 7.0) or in 10 mM Na-Cacodylate (pH 5.0) at increasing concentration of KCl. (**B**, **C**) CD spectra of 4 µM EGFR-272_C acquired in 10 mM Tris pH 7.0 (**B**) or 10 mM Na-Cacodylate pH 5.0 (**C**) with PEG_300_ at 25 °C. Lines correspond to spectra acquired in the absence of crowding agent (red solid line); in the presence of 20% PEG_300_ before (dotted orange line) and after annealing (green dashed line); in presence of 40% PEG_300_ before (blue dashed-dotted line) and after annealing (blue dashed-dotted line), respectively.

The effect of increasing concentrations of K^+^ ions was initially monitored by fluorescence melting assay performed at pH 5.0, 6.0 and 7.0 (Fig. 3A). This set of experiments was performed by using a sequence properly labelled with a fluorophore and a quencher at the 3′- and 5′- ends, respectively (*EGFR-272_C). In the absence of the monovalent cation, a clear thermal transition was detected only at pH ranging from 5.0 to 6.5, with a maximum of thermal stability at pH 5.0 where EGFR-272_C structural equilibrium is maximally shifted from the hairpin towards the iM (Supplementary Table S1).

Upon addition of KCl, different effects were observed according to EGFR-272_C folding state. At pH 5.0, where iM is the predominant species for EGFR-272_C, the presence of the metal ion did not affect the recorded melting profile whereas at pH 6.0 it caused a modest reduction of the melting temperautre (Fig. 3A).

Unexpectedly, at pH 7.0, a well-defined melting transition was detected only in the presence of KCl. The derived melting temperature was directly related to the metal ion concentration and reached a T_m_ of 50 °C at 150 mM KCl. CD melting analysis confirmed the ability of KCl to stabilize oligonucleotide conformation in neutral conditions (Fig. S3). Nevertheless, KCl did not modify the CD spectrum of EGFR-272_C at pH 7.0, thus excluding induction of an iM (Fig. S4). This information further supports that EGFR-272_C arranges into a hairpin form at pH 7.0 which is stabilized by increasing ionic strength. It derives that the iM destabilization induced by KCl in slightly acidic conditions (pH 6.0) is the result of a competition between the iM and the K^+^-driven fold-back structure.

To explore the consequences of the crowded nuclear environment, we titrated EGFR-272_C at pH 5.0 and 7.0 with polyethylene glycol of two molecular weights. Addition of PEG_200_, did not promote iM formation. Conversely, it induced a modest decrease of CD signal and, at pH 5.0, a poor blue shift of the positive band upon annealing (Fig. S5).

A different behaviour was observed upon addition of PEG_300_. Indeed, at pH 7.0 this polymer caused the appearance of a negative peak at 260 nm and a positive one at 288 nm (Fig. 3B). This profile resembles the CD signal characteristic of iM structure. However, the intensity of EGFR-272_C CD spectrum in the presence of PEG_300_ markedly dropped after annealing. This final dichroic profile perfectly matched the one obtained in acidic condition at the highest tested PEG concentration (40%) either in the presence or absence of an annealing step (Fig. 3C). These data suggest that PEG_300_ is able to interact with the oligonucleotide not only when it is arranged in an iM^39^.

### iM of EGFR-272_C efficiently competes with double helix form

**Figure 4:**
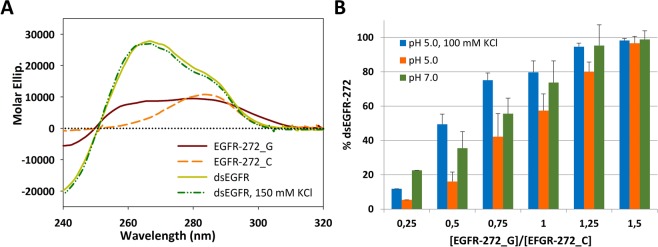
iM formation of EGFR-272_C affects the pairing of the two complementary strands. (**A**) CD spectra of 4 μM EGFR-272_G, EGFR-272_C and of their equimolar mixture recorded in 10 mM Tris, pH 7.0 in the presence/absence of 150 mM KCl at 25 °C. (**B**) Percentage of dsEGFR detected by PAGE from samples prepared at different [EGFR-272_G]/[EGFR-272_C] molar ratio in 10 mM Na-Cacodylate pH 5.0 in absence/presence of 150 mM KCl or in 10 mM Tris, pH 7.0.

Since promoters are double stranded sequences, it was crucial to determine whether the formation of i-Motif by EGFR-272_C efficiently competes with the canonical double helix (dsEGFR). At pH 7.0, an equimolar mixture of the two complementary stands showed an intense CD signal that did not correspond to the algebraic sum of CD spectra associated to the isolated strands (Fig. 4A). The melting profile recorded at 265 nm showed a single transition corresponding to a T_m_ = 71 °C. Moreover, the dichroic spectrum did not change upon addition of 150 mM KCl but the increased ionic strength shifted the melting temperature up to 85 °C. These evidences support the formation of a double-stranded form.

The same was derived by following the pairing of the C-rich and G-rich strands by native PAGE (Fig. 4B). As expected, the electrophoretic band corresponding to EGFR-272_C was converted into a new species at lower electrophoretic mobility corresponding to the double stranded form upon addition of EGFR-272_G. Samples prepared at pH 5.0 clearly showed that the double helix formation was preserved. However, in this condition, the complete pairing of EGFR-272_C required a larger excess of G-rich strand (1:5 molar ratio) indicating that iM can actually impair duplex formation. Interestingly, when KCl was included in the reaction mixture this difference was reverted. This evidence might be unexpected since K^+^ ions induce the G4 folding of G-rich strand that should further compete with duplex formation. However, it is worth to note that the stability of the duplex itself is incremented by increasing ionic strength.

In order to better understand the balance of relative stability of iM, G4 and double stranded form of EGFR-272, additional fluorescence melting analyses have been performed using *EGFR-272_C in the presence of its complementary strand. Data acquired at pH 5.0 in the absence of KCl showed a single melting process (T_m_ = 52.0 °C) (Table 1, Fig. S6A). Upon addition of the metal ion the process was split into two thermal transitions progressively shifted at lower and higher temperatures which were attributed to the iM and double strand denaturation process, respectively. Indeed, as above reported KCl does not affect the thermal stability of iM but instead it stabilizes the double strand form. In agreement with PAGE results, it derives that at equimolar ratio of the two complementary strands, a fraction of the C-rich strand is not paired to the complementary G-rich oligonucleotide.

**Table 1:** Melting temperatures (°C) of equimolar mixtures of G- and C-rich strands of EGFR-272, determined by fluorescence melting assay in 10 mM Na_2_HPO_4_/NaH_2_PO_4_, at pH 7.0 or in 10 mM Na-Cacodylate, pH 5.0 upon addition of increasing concentration of KCl. Errors were ± 0.4 °C.

	[KCl]				
	0 mM	10 mM	50 mM	100 mM	200 mM
pH 5.0 (1^st^)^(a)^	52.4	50	47.5	46.1	44.4
pH 5.0 (2^nd^)^(a)^	52.4	65.5	73.3	77.7	81.6
pH 7.0^(a)^	68.9	74.96	81.7	82.7	84.6
pH 7.0^(b)^	/	74.3	81.0	82.2	83.9

^(a)^Data obtained using the labelled C-rich strand *EGFR-272_C.

^(b)^Data obtained using the labelled G-rich strand *EGFR-272_G.

At pH 7.0, a single transition was progressively shifted at higher temperatures by increasing K^+^ ions concentration. Interestingly, melting temperatures derived by using either the labelled G- or C-rich strand (*EGFR-272_G or *EGFR-272_C, respectively) nicely matched one to each other and allowed attributing them to the denaturation of the double helix (Table 1, Fig. S6B).

### Phenanthroline derivatives efficiently interact with EGFR-272_C

EGFR-272_C appears to be prone to shift among different structures (double stranded, iM, hairpin). Thus, a small molecule able to selectively recognize one of them might represent a useful tool to control this equilibrium and asses its role in physiological processes.

**Figure 5:**
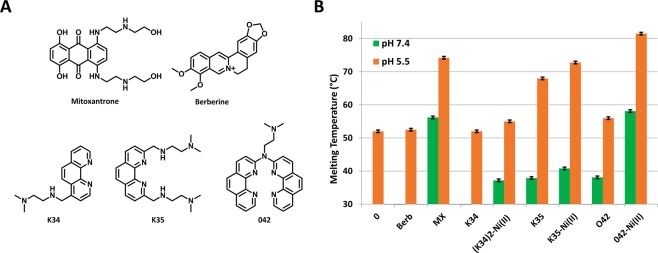
Small molecules significantly stabilize iM of *EGFR-272_C. (**A**) Chemical structures of tested compounds. (**B**) Melting temperature of 0.25 μM *EGFR-272_C recorded in 10 mM Na_2_HPO_4_/NaH_2_PO_4_ buffer pH 5.5 or pH 7.4 in the absence (0) or presence of 2.5 μM ligands.

With this aim we tested three phenanthroline (Phen) derivatives and their corresponding Ni(II) complexes as potential binders of EGFR-272_C iM structure (Fig. 5A). Mitoxantrone (MX) and Berberine have been selected as reference compounds in accordance to their ability to induce and stabilize iM conformation, respectively^40,41^.

All derivatives were preliminarily tested by fluorescence melting assay with *EGFR-272_C at pH 5.5, to verify their stabilization properties on the folded iM and at pH 7.4 to check their ability to induce the iM structure at physiological pH. Results are summarized in Fig. 5B. This preliminary screening highlighted a lower ability of Berberine to recognize or induce the tetra-helical conformation of EGFR-272_C in comparison to MX, in good agreement with previously reported data on other C-rich sequences. Among Phen derivatives, K34 and its (K34)_2_Ni(II) metal complex poorly affected the melting temperature of *EGFR-272_C. Conversely encouraging results were obtained with K35 and 042 that increased EGFR-272_C thermal stability to an extent comparable to MX. Interestingly, at both tested pH, Ni(II) coordination did not significantly alter the behaviour of K35 whereas it largely increased the efficiency of 042.

The most promising iM binders (042, K35 and their metal complexes) were further analysed to acquire deeper insights into their interaction with EGFR-272_C at pH 5.5 and 7.0.

As far it concerns K35, when it was coordinated to Ni(II), it did not cause any changes of the CD profile of EGFR-272_C at both pH conditions (Fig. S7). This indicates that it does not promote relevant DNA rearrangements. The same holds true for K35 at pH 7.0 (Fig. S8A). Consistently, the S1 footprinting of EGFR-272_C in the presence of K35 at pH 7.0 showed the same cleavage pattern of the oligonucleotide alone (Fig. S8B).

**Figure 6:**
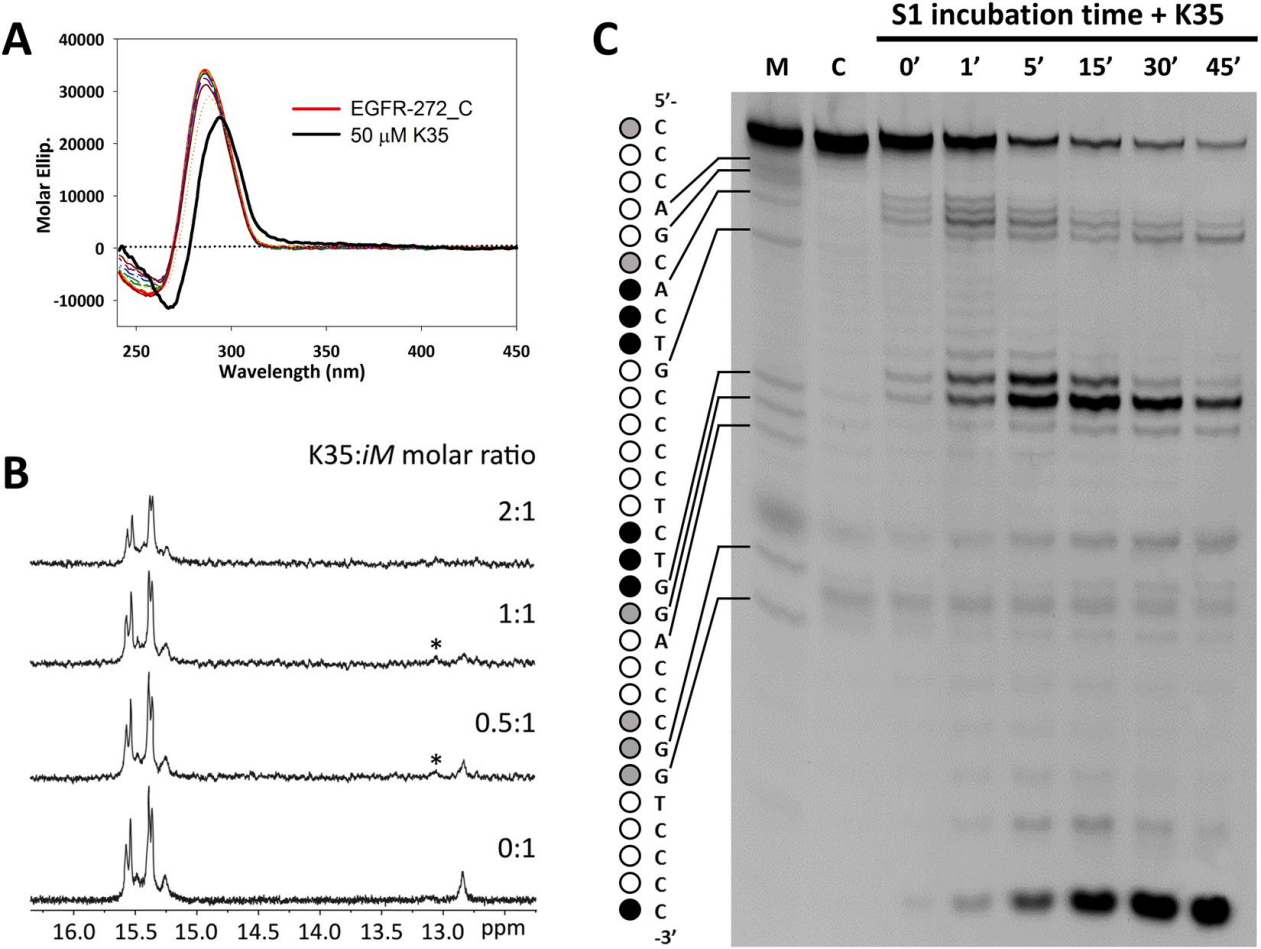
K35 binds different domains of the EGFR-272_C iM. (**A**) CD titration of 4 µM EGFR-272_C with increasing concentrations (0–50 μM) of K35 in 10 mM Na-Cacodylate at pH 5.5. (**B**) Imino region of ^1^H NMR spectrum of 0.15 mM EGFR-272_C acquired at 25° in 20 mM Na_2_HPO_4_/NaH_2_PO_4_ at pH 5.5 in the presence of increasing concentrations of K35; (**C**) S1 footprinting of EGFR-272_C:K35 (1:10 molar ratio) in 10 mM Na-Cacodylate at pH 5.5. On the left side, dots indicate the cleavage sites (black, grey and white dots refer to strong cleavage, moderate cleavage and not cleaved residues, respectively). M refers to Maxam and Gilbert purine marker (purine residues are indicated by arrows on the left side).

A modest variation of the CD spectrum of EGFR-272_C was detected only at pH 5.5, where K35 shifted the positive peak of the oligonucleotide from 288 to 294 nm (Fig. 6A). In order to elucidate the nature of this change, we followed the ^1^H NMR spectrum of EGFR-272_C in the presence of increasing concentration of this ligand in acidic conditions. It is worth of mention that we did not use K35:EGFR-272 molar ratio higher than 2:1 due to NMR signal broadening derived by increasing percentage of DMSO in solution. By adding the Phen derivative, an imino peak at δ 13.06 ppm appeared which was consistent with stabilization of a GC base pair within the iM structure by the mono-Phen derivative. Moreover, at the highest tested molar ratio (2:1), K35 induced a shift (∆δ = 0.06 ppm) of the imino peak at δ 15.49 ppm associated to the protonated cytosine of one C-C^+^ base pair. This suggests an interaction of the Phen derivative with this residue. In agreement, S1 footprinting evidenced that at pH 5.5 K35 reduced the enzymatic cleavage at C6**·**G10 Watson-Crick base pair and at other specific cleavage sites, as G19 and A20 in the second loop and all the residues within the third loop. These data indicate that this ligand recognizes at least one external C-C^+^ base pair of the iM core but also interacts with the Watson-Crick base pairs in the loops.

**Figure 7:**
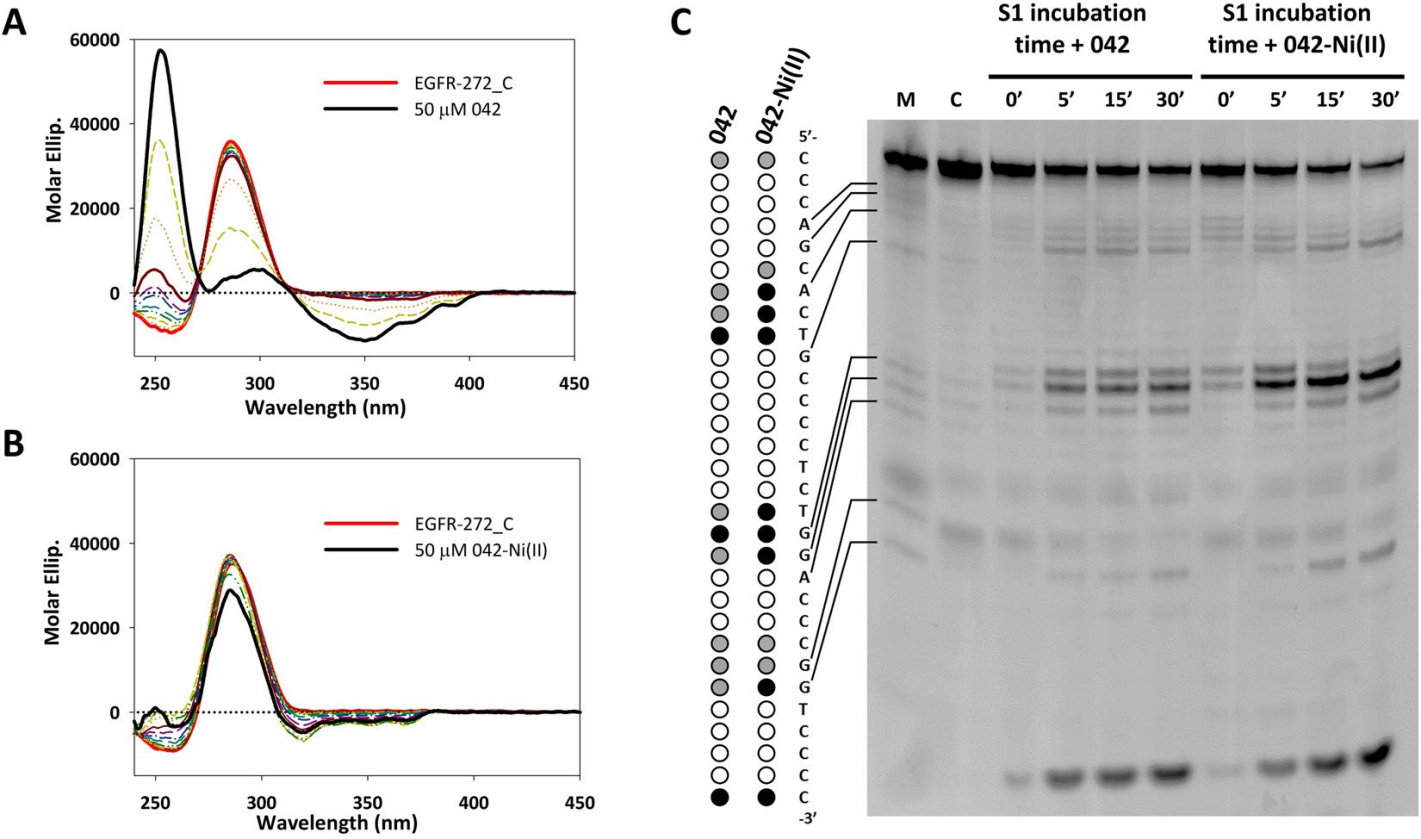
Coordination of Ni(II) alters the binding mode of 042 on the iM of EGFR-272_C. (**A,B**) CD titration of 4 µM EGFR-272_C with increasing concentrations (0–50 μM) of 042 (**A**) or 042Ni(II) (**B**) in 10 mM Na-Cacodylate at pH 5.5; (**C**) S1 footprinting of EGFR-272_C:042 and EGFR-272_C:042-Ni(II) (1:10 molar ratio) in 10 mM Na-Cacodylate at pH 5.5. On the left side, dots indicate the cleavage sites (black, grey and white dots refer to strong cleavage, moderate cleavage and not cleaved residues, respectively). M refers to Maxam and Gilbert purine marker (purine residues are indicated by arrows on the left side).

Also 042-Ni(II) induced small changes to EGFR-272_C CD signal at both tested pH (Figs 7B and S9B). Conversely, addition of the free ligand 042 generated a strong induced CD signal (ICD) with a positive band centred at about 250 nm and a negative one above 270 nm (Figs 7A and S9A). The ICD spectral features at pH 5.5 and 7.4 well overlap one to each other when normalized on the ligand concentration (Fig. S9C) and significantly resemble those previously reported for 042 in the presence of ctDNA or human telomeric G-quadruplex at low ionic strength^33^. S1 footprinting performed at pH 5.5 confirmed a different behaviour of the metal complex vs the free ligand. In particular, 042 significantly protected most of the cleavage sites on EGFR-272_C iM, thus pointing to a not well-defined binding site, in agreement with its recognition mode of dsDNA. Conversely, metal coordination localized the small molecule at the external portions of the structure (Fig. 7C), thus providing a cleavage pattern more similar to the one induced by K35.

## Conclusion

Non-canonical DNA arrangements are interesting potential targets to be exploited in drug discovery projects. However, the preliminary characterization of the structural features of the selected sequences in physiological conditions is a fundamental requirement. Here, we provided the first evidences for the folding of EGFR-272_C into an iM in mildly acidic conditions. Acquired data indicate that it comprises 6 C-C^+^ pairs and three loops of different length: at these domains a network of Watson-Crick pairing provides additional contributions to the overall stability of the iM core.

Interestingly, as reported for its complementary EGFR-272 G-rich strand, we showed that in neutral conditions EGFR-272_C assumes a pre-folded state associated to an intramolecular hairpin.

As a result of this plastic behaviour, under conditions that do not allow complete iM folding (i. e. pH ≥ 6.5) these two arrangements coexist. A shuffling between iM and hairpin–like structures is not novel since it was reported to occur at other pharmacologically relevant sites, i.e. *BCL2* and *KRAS* promoters as well as anti-sense DNA expansions in *C9orf72*^27,29,42^. Worth of mention, environmental factors distinct from pH can significantly contribute in altering it. An example is provided by K^+^ ions that are not able to increase the stability of the iM but are beneficial for the hairpin formation. As a result, KCl can shift the equilibrium towards the hairpin state. Nevertheless, due to the limited thermodynamic stability of this arrangement, in the presence of the complementary strand, the metal ion exerts a global positive role in assisting dsEGFR formation.

Intriguingly, the complex conformational features of EGFR-272_C share extensive similarities with those reported for the G-rich strand. In particular, the two proposed G-C base pairs in the first loop of the i-motif structure of EGFR-272_C (G5-C14 and C6-G10) are potentially conserved also in the hairpin. On the complementary strand, the corresponding residues are localized in the third loop of the two G-quadruplexes formed by EGFR-272 where, as we previously proved, their role is crucial to keep the folding through the formation of two GC base pairs (G20-C21 and G25-C26)^26^. The same bonds are likely occurring in the so called pre-folded states of this G-rich strand. On these bases, the concerted formation of hairpin-like enucleation domains on the two opposite strands might represent a first step to facilitate a coordinated formation of the tetra helical-arrangements when supercoiled removal is required.

In this picture, the search for ligands able to shift the hairpin-iM equilibrium toward one single conformation is attractive. The use of some Phen derivatives as hit for this evaluation provided stimulating hints. We previously showed that G4 structures are preferentially recognized by systems comprising two Phen units organized around one coordinated metal ion according to a planar geometry^31,33^. Taking into account the reduced available surface area of the C-C^+^ pair in comparison to the G4 tetrads, we expected that only one aromatic moiety should be sufficient to target an iM through stacking interactions. The herein collected data turned out to support this starting hypothesis. Indeed, the mono-Phen derivative K35 behaves as an iM binder as efficient as the validated MX. Worth of note this stabilization does not derive only by stacking interactions of the ligand on one C-C^+^ base pair but comprises also a stabilization of the above-mentioned G-C base pairs of the first loop.

On line, the presence of two Phen units in 042 did not provided a synergic contribution to the recognition of the iM. Their role appears to be relevant only in the presence of Ni(II). In this form the planar coordination of the metal ion prevents an extensive not selective recognition of the nucleic acid thus resulting into a implemented iM recognition. This model provides a rationale for the higher efficiency of 042-Ni(II) vs 042 as iM stabilizer.

In conclusion, the results herein presented provide a novel perspective for non-canonical structures with an extremely variegate pool of different arrangements in equilibrium within a short promoter sequence. We need to face this plastic picture as first step for the comprehension of the complex system which attends to the regulation of *EGFR* expression.

## Methods

### Oligonucleotide

DNA sequences were provided lyophilized from Eurogentec (Belgium). They were re-suspended in milliQ water to obtain 1 mM or 100 µM stock solutions. The sequences of oligonucleotides used are shown in Table 2.

**Table 2:** Sequences used in this work.

Sequences	
EGFR-272_G	5′-dGGGGACCGGGTCCAGAGGGGCAGTGCTGGG-3′
EGFR-272_C	5′-dCCCAGCACTGCCCCTCTGGACCCGGTCCCC-3′
*EGFR-272_G	5′-Dabcyl-GGGGACCGGGTCCAGAGGGGCAGTGCTGGG-Fam-3′
*EGFR-272_C	5′-Dabcyl-CCCAGCACTGCCCCTCTGGACCCGGTCCCC-Fam-3′
°EGFR-272_C	5′-CCCAGCACTGCCCCTCTGGACCCGGTCCCC-Fam-3′
22 nts	5′-dGGATGTGAGTGTGAGTGTGAGG-3′
30 nts	5′-dGGGGACCGGGTCCAGATGGGCAGTGCTGGG-3′

### Polyacrylamide gel electrophoresis (PAGE)

The electrophoretic mobility of tested sequences was monitored by 15% native polyacrylamide gel (acrylamide:bis-acrylamide 19:1) in 1x TBE (89 mM Tris, 89 mM Boric acid, 2 mM EDTA, pH 8.0) or 1x TAE (40 mM Tris, 20 mM Acetic acid, 1 mM EDTA, pH 5.0). The pairing efficiency of G- and C-rich strand at both pH (pH 7.0 and pH 5.0) and in presence/absence of 100 mM KCl was examined by using a constant amount of *EGFR-272_C (100 ng) and increasing concentrations of the complementary EGFR-272_G. Before loading, all samples were heated at 95 °C for 10 minutes and then cooled down to room temperature overnight. Solved products were visualized and quantified on a Geliance system (Roche) before and after staining with Sybr Green I.

### Circular dichroism measurement

CD spectra were recorded using 1 cm pathlength quartz cuvette in a Jasco J-810 spectropolarimeter equipped with a Peltier system. Before data acquisition, oligonucleotide stock solutions were diluted to 4 µM in the required buffer (10 mM Tris, 10 mM Na_2_HPO_4_/NaH_2_PO_4_ or 10 mM Na-Cacodylate) and the final concentration was controlled by NanoDrop1000 Spectrophotometer (Thermo Scientific). When required, the pH was changed by addition of HCl solutions. Each reported spectrum represents the average of two scans recorded with a 0.5 nm resolution. The acquired dichroic signal was finally converted in molar ellipticity per residue ([Θ] = deg × cm^2^ × dmol^−1^).

For melting curves acquisition, the variation of the dichroic signal at 288 nm was recorded as a function of temperature from 25 °C to 95 °C at a heating rate of 50 °C/h. The annealing process was acquired as well to check for hysteresis.

### NMR spectroscopy

^1^H NMR spectra were recorded on a Bruker (Billerica, MA, USA) DMX 600 MHz spectrometer, equipped with a 5 mm TXI probe XYZ-Gradient at 25 °C. Sample were prepared in 20 mM Na-phosphate buffer, pH 5.5 or 7.0, 90%/10% H_2_0/^2^H_2_0. Oligonucleotide concentration was 0.15 mM per strand. Suppression of water signal was achieved by applying WATERGATE pulse sequence. Data were processed and analysed by using TOPSPIN 1.3 software.

### S1 footprinting assay

1 µg of 3′-FAM-labelled EGFR-272_C was incubated for 1 h at room temperature in 10 mM Na-Cacodylate pH 5.5 or 7.0. A reaction mixture containing S1 reaction buffer (20 mM Na-Cacodylate pH 5.5 or 7, 300 mM NaCl, 1 mM ZnSO_4_ and 5% glycerol) and 10 (for reaction at pH 5.5) or 20 (for reaction at pH 7) units of S1 nuclease (purchased by Gibco-BRL) was added to oligonucleotide solution at room temperature. S1 digestions were stopped at different incubation time (0, 1, 5, 15, 30, 45 minutes) by adding 6 µl of stop solution (70% formamide, 60 mM EDTA) to 2.5 µl of sample, heating the mixture at 95 °C for 5 minutes and then freezing. Frozen samples were added of 3 µl of formamide and heated at 95 °C for other 5 minutes before loading them on a 20% polyacrylamide denaturing gel (19:1 acrylamide-bisacrylamide, 7 M urea, 1x TBE). The result was visualized on a Geliance system (Roche).

### Fluorescence melting assay

Fluorescence melting curves were recorded in a Roche LightCycler480 (λ_ecc_ 480 nm, λ_em_ 520 nm) in a total reaction volume of 20 µL. Reaction mixtures containing 0.25 µM oligonucleotide were prepared in 10 mM Tris, 10 mM Na_2_HPO_4_/NaH_2_PO_4_ or 10 mM Na-Cacodylate buffers at different pHs. When required samples were added with increasing concentration of KCl (0–200 mM) or ligands (0–20 μM) in the required buffer. The resulting solutions were heated to 95 °C, kept at 95 °C for 5 minutes, cooled down to 30 °C at 0.1 °C/s. After 5 min at 30 °C, data acquisition started during a slow melting step (30–95 °C at a rate of 1 °C/min) and an annealing step at the same rate to check for hysteresis.

To monitor the thermal stability of the double stranded form of EGFR-272, reaction mixtures were prepared by mixing equimolar concentration (0.25 µM) of the C- and G-rich strand (*EGFR-272_C + EGFR-272_G or EGFR-272_C + *EGFR-272_G) in 10 mM Na_2_HPO_4_/NaH_2_PO_4_ at pH 7.0 or pH 5.0. Samples were previously annealed, then the resulting solutions were heated to 95 °C at a rate of 1 °C/s and cooled to 30 °C at the same rate (1 °C/min).

Melting temperatures were determined from the first derivatives of the slow melting profile, using a Roche LightCycler software.

## Supplementary information

supplementary material
