# Supplementary material for: pH-driven conformational switch between non-canonical DNA structures in a C-rich domain of EGFR promoter
Source: Sci Rep. 2019 Feb 4;9:1210. doi: 10.1038/s41598-018-37968-8 (PMC6362134; doi:10.1038/s41598-018-37968-8)
Supplement: Supplementary file 1 — supplementary material [file 41598_2018_37968_MOESM1_ESM.docx]

**Supplementary Information**

pH-driven conformational switch between non-canonical DNA structures in a C-rich domain of *EGFR* promoter

Camilla Cristofari^1,2^, Riccardo Rigo^1,2^, Maria Laura Greco^1^, Michele Ghezzo^1^ and Claudia Sissi^1*^

^1^Department of Pharmaceutical and Pharmacological Science, Padova, 35131, Italy

^2^Authors equally contributed

*claudia.sissi@unipd.it


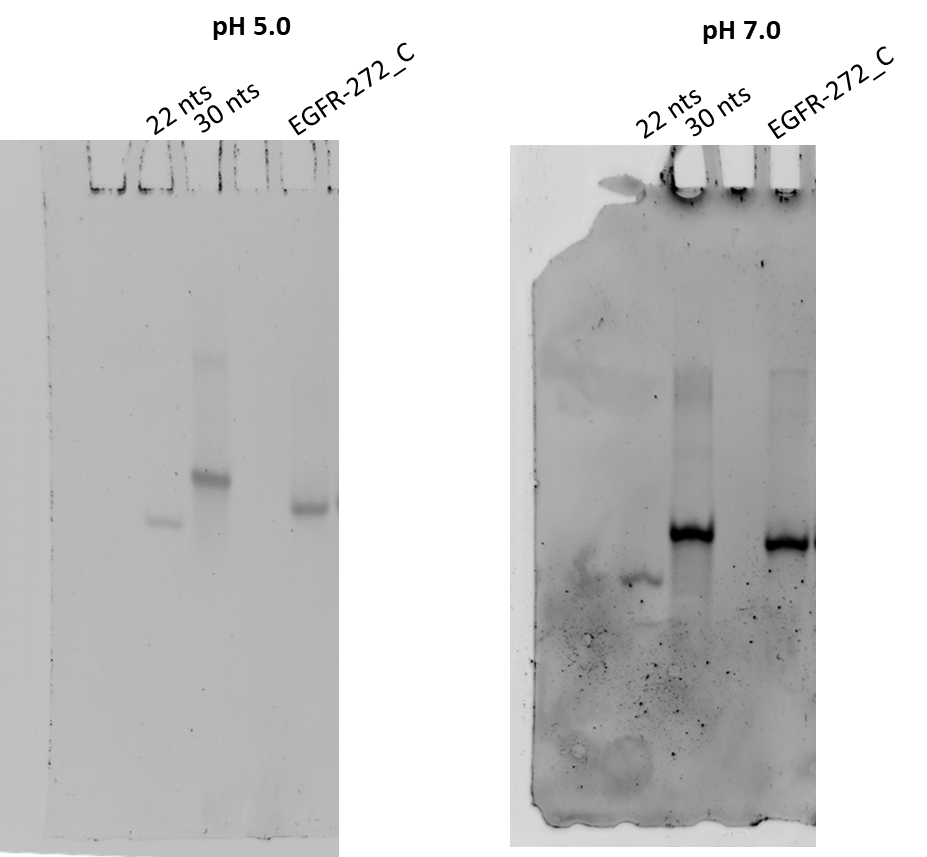


**Figure S1.** Full-length gels of EGFR-272_C annealed at pH 5.0 and 7.0 and solved by PAGE in 1x TBE or 1x TAE, respectively. 22 nts and 30 nts are sequences not forming intramolecular secondary structure under the tested conditions.

**Figure S2.** CD spectra of 2 µM (solid line), 15 µM (dotted line) and 30 µM (dashed line) of EGFR-272_C acquired in 10 mM Na-Cacodylate, pH 5.0 at 25 °C.


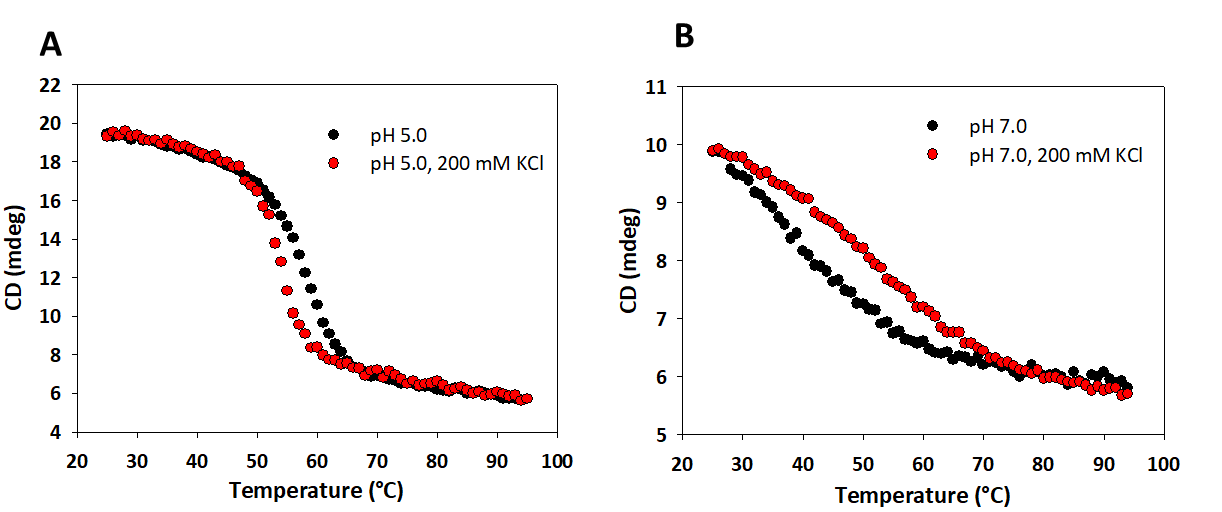


**Figure S3.** Variation of the CD signal of 4 µM EGFR-272_C recorded at 288 nm acquired in (A) 10 mM Na-Cacodylate, pH 5.0 or in (B) 10 mM Na_2_HPO_4_/NaH_2_PO_4_ pH 7. 0 as a function of the temperature in the presence (red dots) or absence (black dots) of 200 mM KCl.


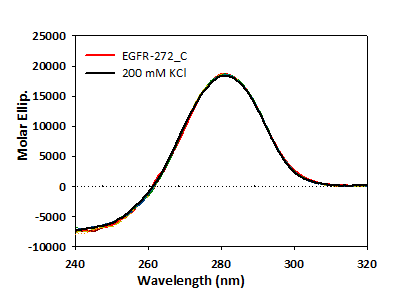


**Figure S4.** CD titration of 4 µM EGFR-272_C in 10 mM Na_2_HPO_4_/NaH_2_PO_4_ pH 7.0 in the absence (red line) or presence (black line) of 200 mM KCl at 25 °C.


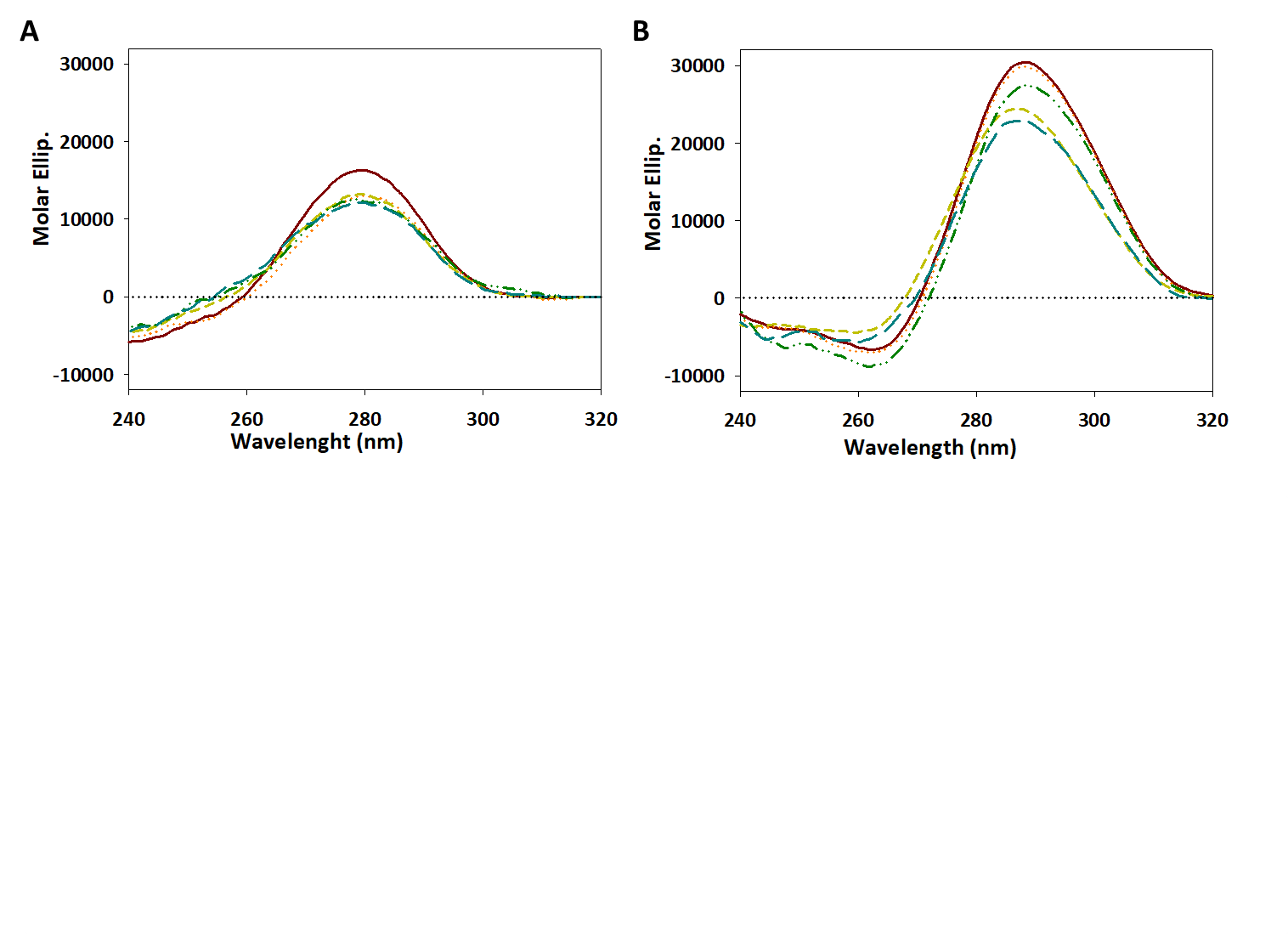


**Figure S5.** CD spectra of 4 µM EGFR-272_C acquired in (**A**) 10 mM Tris, pH 7.0 with PEG_200_ (**B**) 10 mM Na-Cacodylate pH 5.0 with PEG_200_ at 25 °C. Lines correspond to spectra acquired in the absence of crowding agent (); in the presence of 20 % PEG_200_ before () and after annealing (); in presence of 40 % PEG_200_ before () and after annealing (), respectively.


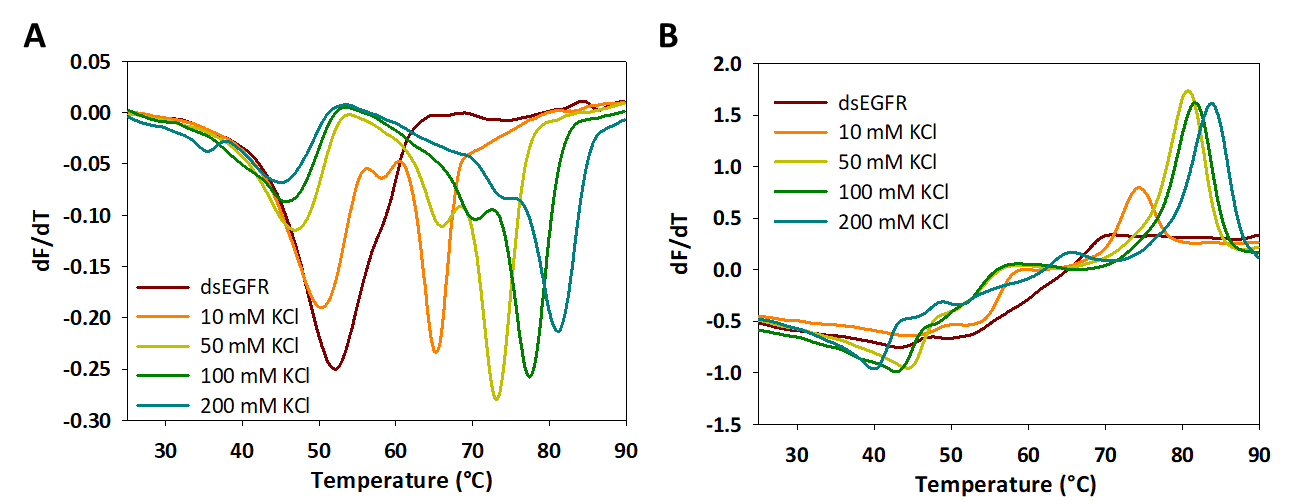


**Figure S6**. First derivatives of melting curves for double stranded EGFR-272 acquired in 10 mM Na_2_HPO_4_/NaH_2_PO_4_ at increasing concentration of KCl by (A) following the signal of the C-rich strand (*EGFR-272_C) at pH 5.0 and (B) the signal of the G-rich strand (*EGFR-272_G) at pH 7.4.


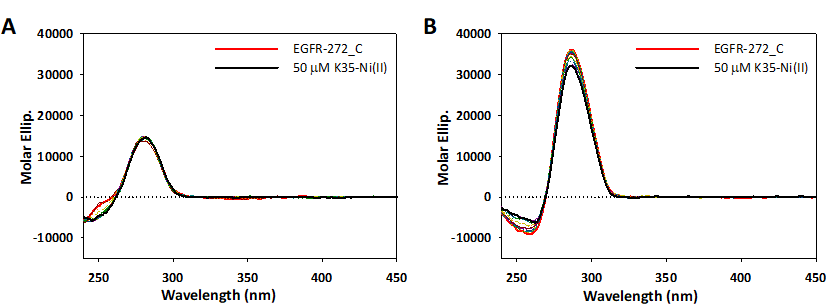


**Figure S7**. K35-Ni(II) poorly affects the dichroic feature of EGFR-272_C. CD titration of 4 µM EGFR-272_C with increasing concentrations (0-50 µM) of K35-Ni(II) in 10 mM Tris pH 7.4 (A) and in 10 mM Na-Cacodylate pH 5.5 (B) at 25 °C.


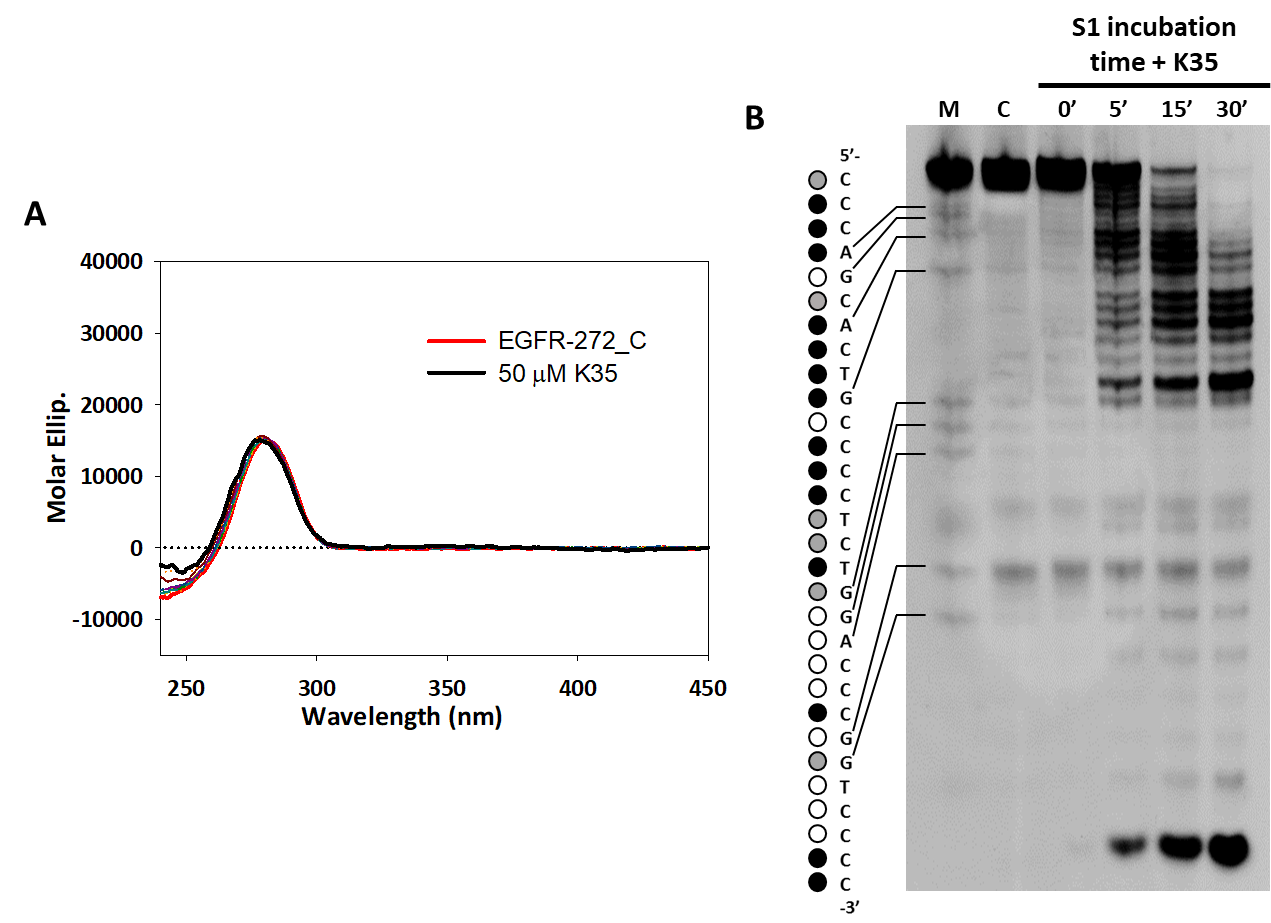


**Figure S8**. At neutral pH, K35 stabilizes the major hairpin form of EGFR-272_C major. (A) CD titration of 4 µM EGFR-272_C with increasing concentrations (0-50 µM) of K35 in 10 mM Tris pH 7.4 at 25
°C; (B) S1 footprinting of EGFR-272_C in 10 mM Na-Cacodylate pH 7.0 in the presence of 10:1 molar ratio of K35. On the left side, dots indicate the cleavage sites (black, grey and white dots refer to strong cleavage, moderate cleavage and not cleaved residues, respectively). M refers to Maxam and Gilbert purine marker (purine residues are indicated by arrows on the left side).


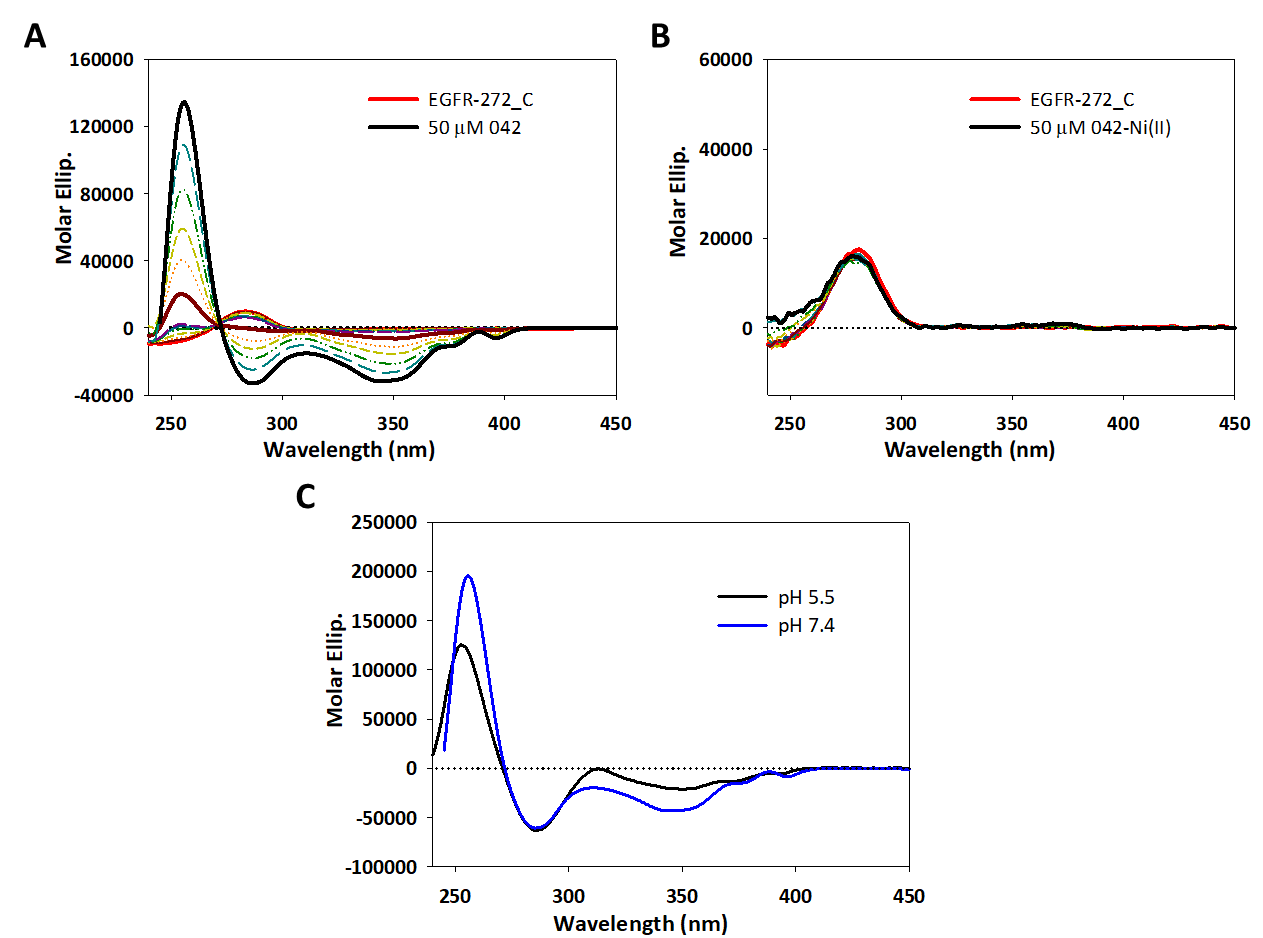


**Figure S9**. Coordination of Ni(II) alters the binding mode of 042 on EGFR-272_C at pH 7.4. CD titration of 4 µM EGFR-272_C with increasing concentrations (0-50 μM) of (A) 042 and (B) 042-Ni(II) in 10 mM Tris, pH 7.4 at 25 °C; (C) differential CD spectra of 042 bound to EGFR-272_C derived at pH 7.0 (blue line) or 5.5 (black line). The molar ellipticity was calculated on the ligand concentration after subtraction of the nucleic acid contribution.
